# Expedited Transition to Digital Delivery of Recovery Support Services Due to the COVID-19 Pandemic: Mixed Methods Needs Assessment

**DOI:** 10.2196/74076

**Published:** 2026-06-30

**Authors:** Tara Karns-Wright, Erin Finley, Robert Ashford, Daniela I Olmos, Ducel Jean-Berluche, Jennifer S Potter

**Affiliations:** 1 Department of Psychiatry and Behavioral Sciences, UT Health San Antonio, 5109 Medical Drive, 4th Floor, San Antonio, TX, 78229, United States, 1 724 816 7645; 2 Be Well Institute on Substance Use and Related Disorders, UT Health San Antonio, San Antonio, TX, United States; 3 Center for the Study of Healthcare Innovation, Impact, and Policy, VA Greater Los Angeles Healthcare System, Los Angeles, CA, United States; 4 Unity Recovery, Philadelphia, PA, United States

**Keywords:** needs assessment, recovery support services, digital recovery support services, peer recovery support services, substance use

## Abstract

**Background:**

Recovery support services (RSS) are an evidence-based approach to support recovery from substance use disorders, most often composed of peer-to-peer support, referrals to housing, job training, and other forms of prosocial engagement and activities. During the COVID-19 pandemic, RSS providers quickly converted in-person services to digital delivery to avoid disruption. It is unclear if this rapid conversion impacted the delivery of services or if this delivery model could enhance RSS reach and uptake more generally by extending the reach of RSS providers and offering an alternative delivery method and access point.

**Objective:**

The goal of this study was to identify how RSS providers in Texas adapted their services for digital delivery and to what extent, if at all, technology limitations (eg, lack of digital infrastructure) were present.

**Methods:**

We conducted an electronic survey of 85 RSS providers, assessing their current capacity and methods for the digital recovery support service (D-RSS), followed by semistructured online interviews with a subset of 20 respondents.

**Results:**

Most survey respondents (74/85, 87.1%) used D-RSS, though they used many dated technologies, devices, and platforms for service delivery. Many respondents indicated that they use Zoom (Zoom Video Communications) videoconferencing to communicate with participants; however, providers also indicated that they must use several different technology platforms to accomplish their service delivery goals. Four main themes emerged from the interviews: (1) the impact of the COVID-19 pandemic on RSS, (2) barriers and facilitators to technology-delivered D-RSS, (3) awareness and expectations regarding the use of D-RSS, and (4) training needs to deliver D-RSS.

**Conclusions:**

RSS organizations have access to technology for D-RSS; however, the technology is often outdated. Because the pandemic required a rapid and unexpected shift to D-RSS to maintain and potentially expand access during a public health emergency, providers desire guidance for training staff and participants on how to best use technology. A subset of providers endorsed the potential of a unified platform for D-RSS delivery, especially for data capture. Most barriers to D-RSS identified by our respondents may be addressable through the streamlined deployment of technology resources, rigorous training and onboarding programs in best practices for providers and participants, and tailored implementation strategies for varying local contexts.

## Introduction

Recovery support services (RSS) are evidence-based approaches [1-3] to support long-term outcomes in recovery from substance use disorders (SUDs). RSSs are distinct from SUD treatment and include nonclinical services that may occur over months or years that traditionally and primarily target cessation of substance use and SUD symptoms. RSSs are ideally delivered by peers with lived or living experience with substance use and recovery in the community, providing individual navigation through the recovery process by someone with personal experience and understanding [3]. These services may include recovery groups, one-on-one engagement with certified peer recovery specialists (peers), and facilitation of other resources such as recovery housing, transportation, education, job training, and physical and behavioral health care referrals [4]. While traditionally these services are delivered face-to-face in a group or individual setting, the COVID-19 pandemic expedited the digital delivery of RSS.

Digital recovery support services (D-RSS) can be delivered remotely using any technology available (eg, text, Zoom [Zoom Video Communications], phone, or a designated recovery support app). Preliminary evidence suggests that D-RSS operates similarly to in-person RSS and can reduce barriers associated with traditional formats, such as accessibility, availability, and cost [2,5]. Research before COVID-19 found that people in SUD treatment are interested in participating in D-RSS, with a preference for a smartphone app or text messaging platform [6], though this study sample consisted of participants from a single northeastern metropolitan area of the United States, limiting generalizability. Despite this, delivery of RSS when the pandemic began, like most health-related appointments and interactions, remained primarily in person with brick-and-mortar facilities. With the need to comply with state-mandated stay-at-home orders, participants were suddenly left without the means to receive services face-to-face and providers without a physical location to work from to deliver services. As a result, there was an urgent need for a rapid shift to delivering RSS digitally. At this point, previous research on D-RSS had not investigated how in-person RSS providers could quickly shift to D-RSS, what technology and infrastructure would be needed by RSS providers, or how services might be impacted.

The Texas state government mandated that the Texas Department of Insurance continue payment parity for D-RSS and in-person RSS for individuals enrolled in state-regulated insurance plans [7]. Large insurance providers followed suit, and RSS-specific changes were also seen in Medicaid reimbursement (eg, audio-only peer services were authorized for reimbursement). While these changes created new opportunities to deliver and receive D-RSS, significant unknowns included accessibility and functionality of devices, technology, and software, in addition to training needs for both providers and participants.

In preparation for adopting D-RSS in a network of recovery providers, Be Well Texas conducted a 2-phase needs assessment to capture adaptations made by providers to meet the demands of a rapidly changing landscape for RSS delivery. Be Well Texas at UT Health San Antonio is a statewide initiative for comprehensive substance use treatment and recovery services available throughout Texas. The objectives of this needs assessment were (1) to identify and describe the technological needs and use of RSS providers and (2) to understand the challenges and facilitators to enhancing the local capacity of RSS providers by leveraging technology and digital platforms.

## Methods

### Study Design

A sequential explanatory mixed methods design [8] was used in this 2-phase needs assessment. The phases were as follows: (1) a survey distributed statewide to 219 RSS providers and (2) semistructured interviews with a subset (n=20) of the survey respondents. The survey data in phase 1 characterized the D-RSS capacity across Texas RSS providers and informed the selection of participants and development of interview guides for phase 2 interviews. The phase 2 interviews then helped to contextualize and further explain the survey findings, with the phase 1 findings providing a structural framework for explaining the themes generated from the interviews [9].

### Ethical Considerations

This project (STUDY00001475) was deemed as not regulated research by the institutional review board at The University of Texas Health Science Center at San Antonio (UT Health San Antonio).

### Phase 1: Survey

#### Participant Selection

RSS providers were recruited via email from the Texas Health and Human Services Commission RSS provider distribution list.

#### Survey Development

A brief survey (see Survey in Multimedia Appendix 1) was developed to assess (1) current device and software use for the delivery of D-RSS and (2) device and software functionality, needed upgrades, and current standard operating procedures for the delivery of D-RSS. Survey items included closed-ended, multiple-choice questions about the current use of D-RSS, access to technology and devices for providing D-RSS (eg, computers, tablets, internet, and software), state of the devices (eg, outdated), and device ownership (personal or organizationally owned). Open-ended questions were used for questions without defined response categories to solicit the full breadth of current RSS technologies and tools used by respondents, what they liked and disliked about them, and an estimate of how many of their participants were receiving RSS digitally. These items were chosen based on identified areas of need by the Texas Health and Human Services and Be Well Texas staff.

#### Procedures

We administered the survey in REDCap (Research Electronic Data Capture [10]), a secure web-based application for building and managing online surveys and databases, hosted at UT Health San Antonio. Texas Health and Human Services and Be Well Texas sent a cosigned introductory email to a listserv of 219 RSS providers to inform them of an incoming survey, to explain its purpose, and to encourage participation. A second email sent by Be Well Texas included a link to the survey and instructions for completion. All providers receive funding to deliver recovery services directly from Texas Health and Human Services, Be Well Texas (as a flow-through grantor from Texas Health and Human Services), or both. Data were collected from December 3‐20, 2021. Respondents were entered into a raffle for 1 of 5 USD $50 Walmart gift cards.

#### Analytic Approach

For the closed-ended, multiple-choice questions, all analyses were descriptive in nature. Categorical variables were summarized using frequencies (n) and percentages (%). For the open-ended questions, an inductive and deductive coding framework was developed for thematic categories. The codes were applied to the open-ended responses, and discrepancies between coders were resolved in regular meetings led by a doctoral-level expert in qualitative methods.

### Phase 2: Semistructured Interviews

#### Participant Selection

We conducted semistructured qualitative interviews with a subset of respondents (n=20 from 16 unique organizations) who completed the survey. Representatives from the organizations were selected based on subject matter expertise and a priori factors that might impact technology access (eg, rural or urban setting and proximity to the US-Mexico border), geographical representation, and population-related factors (eg, age and justice involvement) of the participants served by the organizations.

#### Interview Guide Development

Two semistructured interview guides (see Semistructured Interview Guides in Multimedia Appendix 1) were developed, one for peers actively providing services and one for other types of respondents (eg, leadership at the organizations). Semistructured interviewing allows for follow-up or other clarifying questions when elaboration may be beneficial for understanding, thus eliciting rich detail that a fully structured interview might miss [11]. Interview guides assessed respondents’ use of D-RSS; barriers, facilitators, and expectations regarding D-RSS; and participants’ literacy (general and technological) and comfort with different modes of technology.

#### Interview Procedures

Respondents were informed during the survey that they might be selected for a follow-up interview. In January 2022, we began contacting individuals for follow-up interviews via emails and phone calls. In total, 20 interviews were conducted by 3 trained team members using Microsoft Teams videoconferencing software, with supervision by a doctoral-level expert in qualitative methods. Each interview lasted approximately 45 minutes and was transcribed using the auto-transcription feature in Microsoft Teams, downloaded, edited for transcription errors, and summarized within 48 hours. Postinterview debriefs among the interviewing team were completed immediately following each interview to gather initial insights or concerns. The first 5 interviews were conducted with at least 2 team members present to ensure a consistent approach. The remaining 15 interviews were divided evenly among 3 staff members. All interviews were conducted between January 12 and February 11, 2022.

#### Analytic Approach

We used a combination of inductive and deductive methods in the analysis approach, which provides structured analysis without overreliance on preconceived ideas about what might be observed [12]. The interview data were analyzed using a rapid qualitative analysis approach (under the supervision of a doctoral-level qualitative researcher) to quickly transform data into actionable contributions [13]. Structured summaries were generated from the qualitative rapid analysis and used to determine major themes. These themes were analyzed for subcategories to generate a more fine-grained understanding of D-RSS needs from the interview data.

## Results

### Phase 1: Technology Survey

#### Respondents

**Figure 1.**
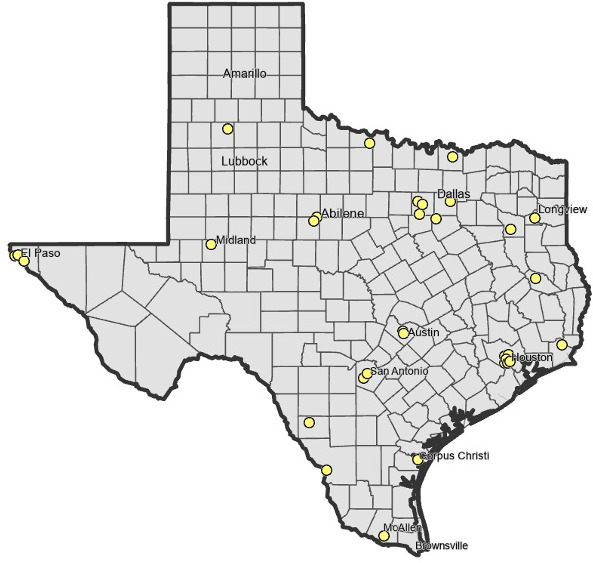
Location of survey respondent organizations (n=34; yellow marker).

We received 85 responses of 219 requests (39% response rate), representing 34 unique provider organizations (Figure 1). Respondents included peer support specialists (50/85, 61%), organizational leaders or substance use treatment professionals (25/85, 29%), and care coordinators (8/85, 9.4%). Respondents served a mix of provider organizations in rural, urban, and border communities, and most organizations delivered RSS across multiple counties, with 1 organization reaching up to 28 counties. Overall, 74 (74/85, 87.1%) respondents were providing D-RSS via various devices and platforms. Yet, 55% (47/85) indicated that “less than half” of their participants engaged with this service modality.

#### Technology Access

Most respondents had access to smartphones (47/85, 90.4%), laptops (39/85, 75%), desktop computers (34/85, 65.3%), and high-speed internet (40/85, 76.9%) for providing D-RSS (Table 1). However, many respondents reported gaps in technology for the delivery of D-RSS, including a lack of high-speed internet (12/85, 23.1%) access and unreliable routers and modems (16/85, 30.8%). Outdated, poorly performing devices—including smartphones (41/85, 48.2%), laptops (35/85, 41.2%), and desktop (31/85, 31.8%) computers (Table 2)—were commonly being used to deliver D-RSS.

**Table 1.** Technology access for digital recovery support service.

Technology	Yes, n (%)	No, n (%)
Smartphones	47 (90.4)	5 (9.6)
Tablets	14 (26.9)	38 (73.1)
Desktop computers	34 (65.3)	18 (34.6)
Laptop computers	39 (75)	13 (25)
Routers and modems	36 (69.2)	16 (30.8)
High-speed internet	40 (76.9)	12 (23.1)
Printers	43 (82.7)	9 (17.3)
Microsoft Office 365	42 (80.8)	10 (19.2)

**Table 2.** Outdated technology (n=85).

Technology	Yes, n (%)	No, n (%)	N/A^a^, n (%)
Smartphones	41 (48.2)	35 (41.2)	9 (10.6)
Tablets	16 (18.8)	12 (14.1)	57 (67.1)
Desktop computers	27 (31.8)	24 (28.2)	34 (40)
Laptop computers	35 (41.2)	32 (37.6)	18 (21.2)
Routers and modems	24 (28.2)	36 (42.4)	25 (29.4)
High-speed internet	26 (30.6)	41 (48.2)	18 (21.2)
Printers	25 (29.4)	48 (56.5)	12 (14.1)
Microsoft Office 365	25 (29.4)	46 (54.1)	14 (16.5)

a N/A: not applicable due to not having access to the technology.

#### Technologies Used for Participant Engagement and Data Capture

Respondents reported using a wide range of technologies for D-RSS delivery (Table 3), with Zoom being the most common (n=30). In total, 18 respondents indicated that they were using a dedicated D-RSS platform. Many respondents reported relying on multiple tools to meet their engagement and documentation needs. For example, 1 respondent indicated using “a number of various spreadsheets in Excel and RecoveryLink” to deliver D-RSS.

**Table 3.** Digital recovery support service (D-RSS) technologies and platforms in use^a^.

Technologies and tools	Type	Function	Values, n
Microsoft Excel or spreadsheet	Documentation	Data capture	2
Salesforce	Documentation	Data capture	2
DigiSigner	Documentation	Data capture	1
RecoveryLink	Documentation, engagement	D-RSS platform	10
Recovery Data Platform	Documentation	D-RSS platform	6
SoberGrid	Engagement	D-RSS platform	2
Clinical Management for Behavioral Health Services	Documentation	Electronic health record, claims payment system	14
Signify Health	Documentation, engagement	Telehealth platform, values-based payment programs	1
Doxy.me	Engagement	Videoconferencing	4
Zoom	Engagement	Videoconferencing	30
Google Duo or Meet	Engagement	Videoconferencing	7
Microsoft Teams	Engagement	Videoconferencing	5
GoTo Meeting	Engagement	Videoconferencing	4
Apple FaceTime	Engagement	Videoconferencing	1
Skype	Engagement	Videoconferencing	1
Smart Recovery	Engagement	Virtual recovery program, meeting locator	1

a Respondents often provided more than 1 technology and/or platform in use. For example, one respondent said, “a number of various spreadsheets in Excel, RecoveryLink,” and another said, “REDCap, CMBHS, Zoom, Doxy.me”).

#### Barriers and Facilitators of D-RSS

Respondents were asked to report barriers and facilitators of D-RSS (open responses). Table 4 provides the coded responses and their frequencies, with broadband connectivity issues and limited participant access to reliable technology as the most common barriers and increased participant access as the most frequent facilitator. Notably, a respondent shared:

*The mobility allows for services to be provided anywhere. This technology is absolutely essential to providing accessible and flexible services that meet the needs of our participants*.

**Table 4.** Respondent-identified barriers and facilitators of digital recovery support service.

Barriers and facilitators	Values, n
Barriers	
Broadband connectivity issues	17
Limited participant access to reliable technology and tools	17
Peer technology literacy	9
Preference for face-to-face interactions	6
Documentation issues	5
Facilitators	
Increased participant access, including overcoming transportation barriers	39
Ease of use	10
Data capture methods	9
Increased area of coverage	6

### Phase 2: Semistructured Interviews

#### Overview

The results of our thematic analysis identified four main themes: (1) impact of the COVID-19 pandemic on RSS, (2) barriers and facilitators to D-RSS use, (3) awareness and expectations of D-RSS, and (4) training needs. Within these 4 main themes are subthemes, all described below. Table 5 provides summary details about the interview respondents, including if they were from a border community, technologies and platforms used for D-RSS, and the populations they primarily serve.

**Table 5.** Interview respondent summary details (n=20).

Organizations (n=16), n	Border community	Interviews, n	Technologies and platforms used for D-RSS^a^	Populations served
1	No	1	Zoom	Adults
1	No	1	Microsoft Teams	Adults and adolescents
1	No	1	Zoom, Microsoft Teams	Adults: house insecure and justice-involved
1	No	1	None	Adults: justice-involved, children
1	No	1	Zoom	Adults: with and without justice-involvement
1	No	1	Zoom	Adults: housing insecure, justice-involved
3	No	3	Zoom, Doxy.me	Adults and adolescents: justice-involved, adults: justice- or CPS^b^-involved
2	No	4	RDP^c^, RL^d^, FaceTime, Sober Grid	Adults: justice-involved
1	No	2	RL, Signify Community	Adults and adolescents: with and without justice- or CPS-involvement
2	Yes	2	Custom Salesforce app; Zoom	Adults: justice-involved, Indigenous community
1	Yes	2	Zoom	Adults: justice-involved
1	No	1	Zoom	Adults and adolescents with and without justice-involvement

a D-RSS: digital recovery support service.

b CPS: child protective service.

c RDP: Recovery Data Platform.

d RL: RecoveryLink.

#### Theme 1: Impact of the COVID-19 Pandemic on RSS

The COVID-19 pandemic prompted a rapid transition to D-RSS statewide due to stay-at-home mandates that forced providers to stop conducting services in person. Initially, respondents reported a successful transition to digital services:

*We’ve been able to reach a lot more people a lot quicker, we’ve been able to maintain contact with them. Before the [pandemic], we’re knocking on doors, sending letters and things like that; suddenly, we get return ...*

[Quote from urban setting]

As the pandemic continued, some technological solutions became burdensome (eg, losing cell phone service), and some participants disengaged with their providers:

*(...) actually, I don’t think there’s a big problem with being able to get reception for Internet or phone services here, but I think it’s more about, you know, either [the participants] are older and they ... All they have is like a small phone, and they can’t see that well on it, or they or they, you know, can’t afford the service for the phone or tablet, and it gets cut off ...*

[Quote from urban border community setting]

Increased access to laptops, tablets, and hotspots from schools and employers initially fostered a smooth transition to digital delivery. However, the shift simultaneously impacted the ability of many participants to discreetly access services:

*We found out very quickly that clients didn’t have any privacy at home. So, they were sitting in their car with their phone doing behavioral health sessions. So, we set up two stations here, so clients could come in and use them ...*

[Quote from urban setting]

Despite these difficulties, the shift to D-RSS was viewed as having generated long-term benefits and remains integral to many organizations, allowing participants to overcome prepandemic barriers, such as long-distance travel, rurality, and lack of childcare:

*I’ve seen more communication, I see more people attending meetings, because they didn’t have to try to make it down to the facility and they didn’t have to worry about catching the bus or gas money or getting off late [from work] to try to make a meeting. So, we’ve seen more people showing up for groups than at any time in the past, because it was just so easy for them to just get on their cell phone. Even if they are on the bus, a bus stop, they’re just getting off of work, and [virtual meetings] made it so much easier. I can see this being a big tool in recovery even after the pandemic, because we’re giving people the ability to be a part of something.*

[Quote from urban setting]

#### Theme 2: Barriers and Facilitators to D-RSS Delivery

Interviewees identified both participant barriers to receiving services and organizational barriers to providing services, with subthemes identified in each. To address these challenges, several facilitators (and subthemes) emerged as well.

##### Participant Barriers

###### Unreliable Internet and Cellular Connectivity

Providers reported that their participants often had issues accessing reliable internet, especially those located in or serving rural areas. Adequate cellular coverage in rural and border communities was also cited as an issue affecting both the RSS staff and their participants:

*In addition, not only the participants have problems with reliable Wi-Fi and internet. I still have staff that don’t have internet. It’s a reality.*

[Quote from rural setting]

*The rural communities have a greater barrier than urban areas because they do not have cell towers.*

[Quote from rural setting]

###### Smartphone Accessibility

Many respondents stated that participants appeared to have access to smartphones:

*But the technology is available. When we started on our harm reduction program about three years ago ... we were going to buy a bunch of phones and just hand them out. But there wasn’t that much of a desire for it, ‘cause they could actually get a free phone somewhere ...*

[Quote from urban border community setting]

However, 5 respondents from 2 organizations (including 2 border communities) mentioned that participants often use pay-as-you-go phone plans or phones with plans that include a limited number of minutes and/or data. Often, participants cannot afford a smartphone or cell phone with an appropriate data plan for accessing high-data consumption apps such as Zoom (Textbox 1).

Textbox 1. **Quotes that demonstrate barriers associated with smartphones and smart devices.**

“... Keeping a smartphone and a computer current is expensive. You know, we have been buying low end phones, but broken phones can get expensive fast. I’m not sure tablets are the way to go. But we’re looking at it” [Quote from urban setting].

“One of the barriers the participants face is [that]changing phones and phone numbers constantly makes it difficult for organizations to reach them” [Quote from rural setting].

“I’m sure that a lot of people would love to have those type of resources—who wouldn’t want to have a tablet too. But some people are barely able to even obtain a cell phone” [Quote from rural setting].

###### Smartphone and Technology Literacy

Though participants may have smartphones, technology literacy for videoconferencing and app use appeared to be low. Several respondents indicated that some of their participants do not know how to effectively use their smartphones for apps such as Zoom and FaceTime.

*A lot of people do have smartphones, but some people don’t know how to use [them] except for Facebook and social media, and they’re very sharp with things like that. But as far as other digital apps, they’re not very savvy with them. Computer savvy, yeah.*

[Quote from rural setting]

Low technological literacy appeared to be most prevalent among individuals older in age. More than half of the respondents from rural communities described generational differences; 3 of the 4 respondents representing border communities shared this perspective as well. Without assistance, older participants sometimes have difficulty downloading an online app or are unwilling to download an app or work with other technologies outside their comfort zone.

*I think that [things] like installing apps on their phones, it’s just a little bit harder for the older clients, because we’ve had issues [with that].*

[Quote from urban border community]

*When we do get back to face to face, we have to have a hybrid so that the physical presence [is there] but being also technologically connected so that everybody can participate. That was a great find for us during the pandemic because the younger people, they love it. They thrive in that environment and that’s what they want and they can feel as connected as an older generation that doesn’t feel connected electronically. They want face to face and you can meet both needs and that’s what’s important. Everybody has different needs and different barriers.*

[Quote from rural setting]

Literacy barriers were also identified for justice-involved RSS participants, specifically, for those experiencing extended periods of incarceration:

*... I work a lot with people who come out of prison, and [maybe] they’ve been in prison for a while. All of a sudden, we have iPhones and they’re like “What do you do with this thing?” And I’m like, “well, it’s just like a computer” and they’re like “well, I haven’t been around a computer. I don’t know how to work at computers.” So, it’s really difficult for them to be able to say, “Hey, I need to download this app. And how do I work it?” It’s pretty simple for me ... but for somebody who’s fresh out of prison and hasn’t seen a phone in 10 years, then they’re really shocked about it.*

[Quote from rural setting]

###### Housing Insecurity

In total, 16 respondents served participants experiencing housing insecurity, most reported in urban areas. One respondent estimated that up to 90% of their participants had recently, or were currently, not residing in a permanent living space, which creates difficulty in communicating with participants.

*... if they can’t get [to] a program, what we do to try to alleviate that is ... we ask them, “where do they hang out? What store? What streets?” So that if we can’t get a hold of family members, we can at least go out and look for them ...*

[Quote from urban setting]

###### Language and Literacy

Respondents reported that most participants they have served were fluent in English, though 3 organizations also worked with Spanish-speaking individuals, comprising up to 75% of their participant population, and were unable to provide services in both English and Spanish to meet this demand:

*We don’t have Spanish language or Spanish-English bilingual coaches on staff and we have several volunteers. One volunteer is not bilingual, she’s Spanish only. And so, we are working at expanding that out and are looking at that increasing over the next year.*

[Quote from urban setting]

*It could take you an hour to enroll somebody do the recovery capital, do the assessment, do the recovery plan. And if it takes an hour doing it in English, it takes that much longer to do it in Spanish and translate and sometimes you’re having to toggle between “Google translate” a lot.*

[Quote from rural setting]

Furthermore, respondents from 4 organizations stated that approximately 50% of the individuals they serve have reading literacy challenges—their participants often had difficulty understanding documents related to their care and the services they are receiving. For example, one respondent mentioned:

*They accept anything that’s told to them, they sign anything that’s put before them without understanding, without questioning. Now, even if they were not to agree, they would look at it as detrimental to them. Because somebody would then ... look at him in a different way that makes it harder to get through their probationary period.*

[Quote from urban setting]

###### Importance of Face-to-Face Interactions

Despite the frequent use of D-RSS in this study, several respondents (n=8) from organizations serving primarily urban communities mentioned the importance of delivering RSS to participants in person. They described how efforts to engage with individuals in the field often center on building trust and engagement with the services:

*We don’t just have them [participants] come to us and sit in our office. We go to them a lot and that’s I think one of the reasons (...) we had a lot more connection with our people long term than a lot of the other programs.*

[Quote from urban setting]

*We’re talking to people out in the streets, and so they know our face and they know our numbers. So they call us and tell us when there’s a relapse ... I wanna say 80% of the time they’re calling up peer recovery before they call the cops.*

[Quote from urban border community setting]

Respondents noted that for some participants, the sense of trust developed by being embedded in the community may not happen as organically in a digital relationship:

*... A lot of people are really buying into more digital ways of communicating and more ways to access services remotely. I think that it, of course, will present challenges because you have people in their own way of thinking like “no this this can’t be right: how could I possibly like really connect with this person via this online platform?”*

[Quote from urban setting]

Finally, while videoconferencing has allowed organizations to expand services, especially into rural areas by removing barriers such as transportation and childcare, one respondent acknowledged that D-RSS, even with video interactions, will not always allow them to thoroughly assess a participant’s well-being:

*You can’t really use all your senses to see how your client is doing through Zoom. It’s just their smile, their face and that’s it. But when you see somebody face to face, you could see different stuff. Like the clothes they are wearing, if they’re tattered, if they haven’t taken care of themselves, if they smell], stuff like that.*

[Quote from urban border community]

###### Preference for Phone Calls and Text Messaging

Many organizations are providing D-RSS via phone calls and text messages. Respondents representing organizations serving urban communities shared this preference significantly more than their rural counterparts. Texting appears to permit respondents to quickly engage with participants with a higher response rate:

*I have one recovery coach who every morning sends an individual text of encouragement to every one of his clients. Even after they drop off, he continues, and then that sometimes brings them back because they know somebody is thinking about them and cares about them ... but if they had to open a special app to see it, I don’t know if they would.*

[Quote from urban setting]

*It’s amazing how [much] quicker of a response you get via text message, than a phone call or anything else.*

[Quote from rural setting]

### Organizational-Level Barriers

At times, staff members take on multiple roles for their organization to be able to meet the existing demand for services. In total, 5 respondents mentioned low staffing as a barrier to providing services to individuals in general. One respondent said:

*We need more workers, plain and simple. We need more, the need is so big, so great. We think we are doing a great job ... but even in that we’re still missing.*

[Quote from urban setting]

Although the inclusion of D-RSS could address some of these bandwidth issues, multiple organizations still mentioned staffing as an issue to optimally using D-RSS platforms:

*... Even if we had the transportation and we had all these tools, I lack manpower to be able to do what we can—like more education groups, wellness groups, social activities, all of that could happen. If I had more people, for example, when we had those large groups on Zoom, I could have split those up and not have so many people where you can’t really participate. If I have more coaches, we split up different groups. Everybody takes 10 to 15 [participants] and then you have a more manageable group.*

[Quote from rural, border community setting]

### Facilitators: Participants and Organizations Providing Services

#### Champions

In total, 7 respondents, all from urban settings representing distinct organizations, including 2 border community organizations, described employees who went above and beyond to ensure that their participants remain engaged in D-RSS. Employees might arrive at work early to set up Zoom meetings, remain attentive to attendees who get disconnected, and thoroughly follow up with them if participation decreases. One organization even conducted an online survey with its participants to ensure that Zoom would work for them. The commitment is best captured by this interviewee:

*Everybody is in recovery from alcoholism or drug addiction and we’re just on fire to serve that new person that seeking recovery.*

[Quote from urban, border community setting]

#### Staff Comfort With Technology

Both rural- and urban-serving respondents reported a high level of comfort with features such as texting, phone calls, navigating to websites, and smartphone use in general:

*All of our coaches ... have extremely high aptitude in technology, it’s one of the things we screen for during the interview process.*

[Quote from urban setting]

*All of our recovery peer support specialists are very familiar with all the platforms, and they are very comfortable with using them.*

[Quote from rural setting]

#### Openness to Digital Delivery

In total, 13 of the 16 organizations represented by respondents indicated that they would be open to using D-RSS, one highlighting:

*... Our organization, and I could probably speak for most organizations from El Paso ... [we] are waiting for somebody to come and show us ... what’s available. I talk to a lot of recovery coaches throughout the city and a lot of them are on the same boat as we are. We started off big with Zoom, but then it ... teetered off. It would be great [if] somebody comes along and tells us “Pick what best works for your organization.” That would be awesome.*

[Quote from urban border community setting]

Yet, the need remains to “... provide equipment for both the participants and the RSS specialists” to ensure access for the successful use of these tools. A respondent from the same organization spoke about a hybrid RSS set-up where participants could opt for D-RSS or in-person RSS:

*[We] would like to have a drop-in center where [the participants] can come and have access to the services and it does not mean they have to come in person, but they can just come and access the services they need.*

[Quote from rural, border community setting]

#### Ease of Use for RSS

Ease of use for D-RSS platforms was also an essential facilitator for RSS organizations, at times made possible by having the right equipment:

*... We’re given laptops that we can take to and from home and we’re given portable Wi-Fi. So we pretty much can get in contact with any of our participants or clients at any given time whenever needed.*

[Quote from urban setting]

### Theme 3: Awareness and Expectations of D-RSS

#### Awareness and Current Use of a D-RSS Platform

Awareness and use of unified D-RSS platforms for engagement and documentation varied widely across organizations. Only 6 respondents representing 3 organizations (all in urban settings) reported using a dedicated D-RSS platform. Most organizations instead relied on multiple disconnected technologies and software, such as smartphones, videoconferencing platforms, state electronic health records, and spreadsheets, to meet service and documentation needs.

Of the organizations using a D-RSS platform, one in an urban border community was using a version of the Salesforce platform that they had customized specifically for D-RSS delivery. In total, 4 respondents from 2 organizations described participant-centered customizability and availability of options for delivering and recording delivery of RSS as key features. Other features were described that would improve a D-RSS platform:

*... [to]generate little graphs every time you meet with a peer; you could ask five questions about different points, different pieces of recovery capital [recovery capital is a measurement of a participant’s resources that can be used to initiate or maintain recovery], or different parts of recovery, or quality of life, and then how they feel about the service that they received from the individual that is providing coaching ... Recovery Link [the platform used] doesn’t have that*.

[Quote from urban setting]

#### Hybrid Capabilities of D-RSS Platforms and Training

Respondents from both rural and urban settings indicated that an RSS platform that could be used for hybrid services (digital and in-person) would be beneficial for their organizations.

*The key is to serve people where they are at, if they need face-to-face then we do face-to-face or virtually.*

[Quote from urban setting]

#### Documentation and Reporting Capabilities of D-RSS Platforms

Respondents highly valued the ability of D-RSS platforms to document interactions with participants, including the ability to generate reports. Respondents from 14 organizations (5 rural and 9 urban, including 2 border communities) mentioned double documentation of services as an issue stemming from different grants or contracts requirements. Therefore, the ability to upload all required documentation to one platform was highly desirable. One respondent saw the potential benefit of D-RSS platforms to:

*... cut down on some of that documentation to where we could better serve the person’s needs, instead of tracking them down and sticking a stack of paper in front of them or having to get hold, to try to get them to sign something.*

[Quote from rural setting]

Furthermore, one respondent emphasized that documentation can also facilitate building rapport between the providers and the participants:

*Our coaches use RecoveryLink more for data input than anything else ... because we really lean into personal rapport building, RecoveryLink is generally not even something that’s open during a session ... our peers are encouraged to document after a session.*

[Quote from urban setting]

In total, 7 respondents indicated that data capture and reporting are beneficial in a D-RSS setting due to the current options. A total of 3 respondents mentioned challenges using the state data platform for generating reports, citing double documentation and limited training availability as specific challenges. A total of 4 other respondents indicated that their organizations use multiple programs for reporting, which can become cumbersome and vulnerable to error.

#### Funding

Respondents from 12 organizations (5 rural, 7 urban; including 3 border community settings) stated that they need external funding to support a transition to a D-RSS platform. In total, 5 respondents (including 5 from border communities) spoke about needing additional funding to hire a staff member to conduct training sessions for their peers and participants and uncertainty about how they can afford to do so. A total of 3 respondents mentioned that access to appropriate technology (ie, smartphones, tablets, and computers) for both participants and peers is one of the biggest barriers to providing D-RSS:

*The company doesn’t give them ... like laptops or tablets or cell phones, so they’re having to work from their own [devices].*

[Quote from urban setting]

One respondent mentioned that current funding restricts them from purchasing equipment for their peer support specialists and participants.

*... more funding opportunities that designate purchasing telephonic equipment or telecommunications equipment for individual participants specifically like the Be Well VBH program that’s accepting applications. If there were more opportunities like that, it would change the landscape.*

[Quote from urban setting]

*... I can see that being a definite challenge if we’re gonna need to ... supply devices to individuals we serve. Funding might be the only thing [preventing us from providing] devices to our clients ... I don’t see any other real barriers.*

[Quote from rural setting]

### Theme 4: Training Needs

All except 3 respondents (including 6 rural and all 4 border community settings) mentioned training as a major barrier to D-RSS uptake:

*If we could train them [participants] on how to use it. And ... if we provide them with the equipment they need ... I think if they have the equipment ready, and the training, then I don’t see why it would be a barrier. But the biggest thing would have to be the training.*

[Quote from rural setting]

Concern for adequate training was mentioned considerably more in respondents serving rural and border communities compared to urban communities:

*Realistically, I definitely think you need to do a training component to build that confidence for it to be successful. But I think in order for it to be successful, you’d have to have a checklist of making sure all the equipment is in place, the training is in place, the comfort level is in place.*

[Quote from rural border community setting]

An additional desire was the availability of technical assistance since not everyone learns at the same rate:

*With any new thing, what we hear from our staff the most, is “don’t just throw me some new software, give me a little bit of training on it.”*

[quote from urban setting]

While many respondents mentioned working with participants who had high technology acceptance and technological literacy, others described challenges in helping participants to learn the virtual platform and their subsequent decision to offer hybrid training meetings to meet the needs of different generations:

*Typically, if they didn’t know how to use it, we would give them a YouTube link to show them how to maneuver through Zoom and what they had to do. It was more difficult with the older population ‘cause they still have flip phones, and they didn’t have smartphones. That made it a little more difficult. But if they still needed help, they could even come in in person and we could show them what they needed to do, and help them set up their phones.*

[Quote from urban setting]

Conversely, respondents serving urban communities, including one border community, described having a 1-week-long training course and other training throughout the year, including onboarding training for new staff and ongoing training as part of contract requirements. For one organization, training included sessions to understand how to document the delivery of services and export data for reporting.

## Discussion

### Principal Findings

The objectives of this needs assessment were (1) to identify and describe the technological needs and use of RSS providers and (2) to understand the challenges and facilitators to enhancing the local capacity of RSS providers by leveraging technology and digital platforms.

Our phase 1 survey highlighted that most RSS organizations (n=74, 87.1%) were using D-RSS but relied on multiple types of technology, devices, and software to perform service delivery. The phase 2 interviews revealed four main thematic findings: (1) COVID-19 had a significant impact on RSS delivery, with most respondents indicating that they swiftly transitioned to digital delivery despite limited preparation; (2) there were identified barriers and facilitators to D-RSS at the participant level (eg, unreliable internet and cellular connectivity) and the organization level (eg, staffing issues and a high openness to D-RSS); (3) varying awareness and expectations of D-RSS, such as the desire to have a unified D-RSS platform that allowed for participant communication and documentation, but the need for additional funding to purchase and implement such a platform; and (4) high training needs due to low technology literacy among participants.

### Considerations for Delivery of D-RSS

Some recovery support settings may not be suitable for D-RSS. For example, organizations that serve individuals who are incarcerated or those that require intensive, in-person support (eg, help with apps or accompaniment to legal services and download a phone app) continue to rely on face-to-face interactions. Some participants, particularly older adults, also report greater comfort with in-person engagement. For these reasons, hybrid models that offer both digital and in-person options may be optimal.

Additionally, the phase 2 interview data elaborated and explained several patterns observed in the phase 1 survey data. For example, there appeared to be a disconnect between the availability of technology and the capacity to use the technology, highlighting that access alone is not sufficient without the infrastructure, training, and organizational support to learn it. Our respondents indicated that participants’ comfort with technology varied widely, with most being able to engage digitally, but challenges were common among older adults and individuals with limited experience using smartphones or videoconferencing (eg, individuals who had been incarcerated for an extended time). Often, these challenges can be overcome with training, resource allocation, and technical support, which was a common theme in the interviews.

These findings are consistent with prior research that found high smartphone ownership (93.8%) in SUD treatment settings, though many individuals had monthly phone plans with limited data and significant age differences in how smartphones and social media were used [6]. Together, these findings and the findings of our needs assessment suggest that access to technology alone is insufficient; but rather, the type of access, affordability of the data needed to use the technology, and user comfort with the technology all affect D-RSS.

In fact, while respondents were generally open to using D-RSS, their training needs differed by setting. For instance, urban settings were more likely to describe barriers such as participants experiencing housing insecurity; respondents in rural settings were more likely to describe barriers related to broadband coverage. While needs in US-Mexico border communities often reflected those of rural communities, not all these needs overlapped consistently. Tailored training approaches may be necessary to meet the needs of participants and providers with varying levels of technology literacy.

Several respondents preferred face-to-face, in-person RSS delivery over D-RSS because they could more fully assess how a participant is doing (eg, personal hygiene). However, it is important to note that as providers of RSS, these preferences are situated specifically within the provision of RSS, not the receipt of RSS; importantly, participants receiving RSS may have similar or varied preferences. Other respondents preferred to use cell phones to call and send text messages to participants. However, we note that it is unclear whether there are safety and/or privacy issues using text messages for D-RSS in this manner, given that SMS platforms are not traditionally secure and encrypted end-to-end [14]. This speaks to the relatively nascent nature of D-RSS as well as the organizations providing them. These findings collectively highlight the importance of our recommendations for additional, robust D-RSS platforms and provider training. Additional training could directly address how to make D-RSS delivery more like in-person sessions and include additional content to overcome perceived limitations of D-RSS delivery. Likewise, training programs could emphasize the seamless and easy-to-use nature of voice and text messaging features of a D-RSS platform, particularly if one platform is used across settings.

### The “Digital Divide” and D-RSS

As of the most recent assessment by the Texas Broadband Development Office in June 2022, 2.8 million Texas households and 7 million people do not have access to high-speed broadband, and 23% of Texas people could not attend a telehealth appointment from home. Of the households with broadband access, 5.6 million households do not have a reliable internet connection [15]. This situation limits both their access to in-person RSS and their likelihood of connecting to D-RSS. In March 2024, the Federal Communications Commission voted to update the minimum standards to 100 Mbps download speed and 20 Mbps upload speed (increased from 25 Mbps download and 3 Mbps upload) [16].

**Figure 2.**
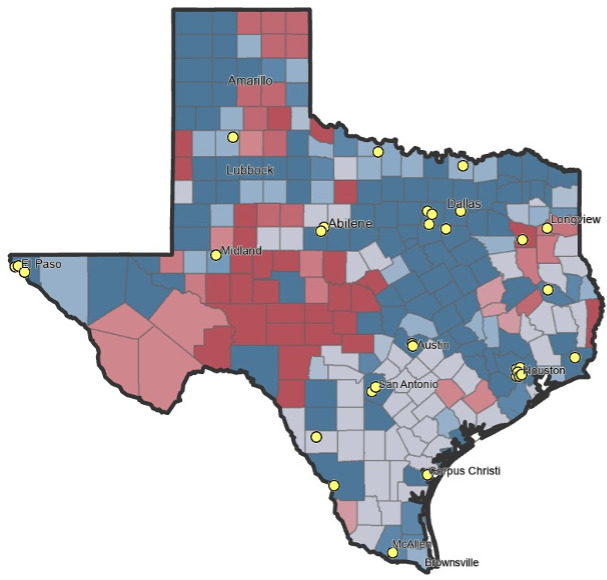
Locations of survey respondent provider locations (n=34; yellow marker) and broadband access. Data as reported from the Federal Communications Commission, June 2021.

To document what some respondents reported about connectivity, we mapped all known RSS provider locations and broadband connectivity (as a function of speed of the broadband) throughout Texas (Figure 2). Areas in gray and red are where broadband speeds are at or slower than 25/3 Mbps; all are below the recommended 50/5 Mbps for telehealth. Furthermore, we duplicated this map using cellular towers and cellular connection availability, resulting in a very similar pattern; however, broadband is recommended for telehealth services due to its increased reliability compared to cellular connections. Figure 2 demonstrates substantial areas in Texas that do not have the appropriate broadband speed to support virtual services.

The structural barriers documented in this needs assessment, such as outdated devices and limited broadband infrastructure, are not only logistical inconveniences but also speak to the broader differences in nonmedical drivers of health experienced by Texas people. White’s [3] model of community resource mobilization for SUD recovery emphasizes that building community capacity, not simply connecting people to services, is essential to sustained SUD recovery. The findings here suggest that implementation strategies for D-RSS must also consider the community conditions (eg, broadband coverage and affordable cellular data) to determine if D-RSS is truly accessible.

### Effects of the COVID-19 Pandemic on RSS

The COVID-19 pandemic accelerated the adoption of digital tools such as smartphones, computers, Zoom, FaceTime, RecoveryLink, and the Recovery Data Platform for D-RSS. These digital tools enabled RSS providers to maintain contact with participants, overcome geographic barriers, and expand access to RSS for those who have struggled to maintain engagement. As D-RSS becomes more embedded in RSS practices, a unified platform for documentation, communication, data capture, and participant engagement may help streamline future implementation, training, and evaluation efforts. The rapid and unplanned transition of RSS to D-RSS examined here suggests that many organizations were able to leverage the potential reductions in barriers to accessing in-person RSS mentioned previously, such as accessibility and cost [5]. However, the improvements in recovery capital with sustained and structured peer engagement documented in existing research [2] emphasize the need to maintain consistent and high-quality peer-to-participant relationships while capitalizing on reductions in access barriers that the COVID-19 pandemic made visible.

### Distinction of Virtual RSS and D-RSS

While virtual and digital options for the delivery of any intervention, including RSS, are often comingled, we feel it is important to highlight that there is an important distinction between the classification of virtual RSS versus D-RSS and the definitions of such varied delivery channels. As described in the literature [3-5], D-RSS are delivered digitally in 2D fashion, largely through video screens and telephones. Virtual RSS is instead delivered in 3D spaces and is only recently emerging in the field in the last 2 years through metaverse communities such as the RecoveryVerse [17,18]. Future research should pay particular attention to establishing emerging empirical evidence on RSS delivered virtually, as there is scarce literature to date and only emerging evidence on D-RSS.

### Limitations

As with any needs assessment, this work has limitations. The total sample of respondents (n=85, 39%) is a small proportion of the total number of individuals involved with the delivery of RSS in Texas. The sample of providers selected for the semistructured interviews was also small (n=20). However, in both cases, efforts were made to solicit responses from diverse providers in a variety of positions or roles within RSS organizations and locations across Texas. We conducted qualitative interviews with individuals who could provide diverse perspectives on many topics related to D-RSS use and expectations. However, we acknowledge that our sample is not likely representative of RSS providers in Texas, and interpretations based on these data must consider these small sample sizes. Additionally, due to the nature of the survey, we do not have information that explains why respondents could not access specific technology in addition to their specific demographic characteristics. This lack of information should be considered when interpreting the results, and we recommend these as future areas of study and analysis.

### Conclusions

In this study, the integration of quantitative survey findings and qualitative interview data resulted in insights that neither method alone could have produced. For example, the survey demonstrated access to technology and D-RSS use across Texas, while the interviews revealed some of the mechanisms, experiences, and contextual nuances behind the access and D-RSS use, including how RSS providers navigated the rapid digital changes, why certain technologies and digital platforms were preferred, and what infrastructure gaps were present (eg, training and education and broadband coverage). Implementation strategies to facilitate the adoption of D-RSS in Texas should be based on a systematic assessment of community needs and local context, such as an assessment of the technology and digital tools and the appropriate infrastructure to support them. Overall, the COVID-19 pandemic revealed both the resilience and the fragility of the RSS in Texas. To fully realize the benefits and potential of D-RSS, community-level infrastructure such as broadband equity, technology and digital device access, and workforce development and training requires dedicated improvements. Future research should examine these community-level factors across time to speak to the sustainability of D-RSS in Texas.

## Supplementary material

10.2196/74076 Multimedia Appendix 1 Phase 1 survey and phase 2 interview guides.

## Abbreviations

D-RSS: digital recovery support service
REDCap: Research Electronic Data Capture
RSS: recovery support service
SUD: substance use disorder
