# Supplementary material for: Expedited Transition to Digital Delivery of Recovery Support Services Due to the COVID-19 Pandemic: Mixed Methods Needs Assessment
Source: JMIR Form Res. 2026 Jun 30;10:e74076. doi: 10.2196/74076 (PMC13318200; doi:10.2196/74076)
Supplement: Multimedia Appendix 1 [file formative-v10-e74076-s001.docx]

**Multimedia Appendix 1**

**S1. Survey**

As part of broad effort to better understand the technology needs for recovery support services, HHSC is contracting with TxMOUD [Be Well Texas] and UT Health San Antonio to survey recovery service support providers and youth recovery centers on this topic with the ultimate goal of reaching more individuals in need across our state.

Your participation in this survey is highly encouraged and instrumental in improving the technology infrastructure to support a robust statewide crisis response capacity delivering and documenting RSS and YRC services and outcomes, and, ultimately, saving lives through recovery support.

Thank you for taking this survey!

*Organization and respondent role in organization*

Please answer the following questions:

1. Please provide the name of your organization: ________________
2. Are you a peer support specialist?
   - Yes
   - No
3. *[If answered no to Question 2]*: What is your job title? Please include your profession and educational degrees.

_______________________

*Contact information for semi-structured interviews*

To better understand your organization's technology needs, we may want to conduct an interview with you or someone in your organization.

Please provide your name and contact information, or the name and contact information from someone in your organization for a follow-up interview.

1. Full name: _______________
2. Phone number: _______________
3. E-mail address: _______________
4. Physical address (of your organization) : _______________
5. Please describe the participant population you serve (e.g., adolescents, individuals that are incarcerated or recently released, individuals in medication-assisted treatment, etc.):

__________________________________________________

*Technology access*

1. *[If respondent indicated they are a peer support specialist]* Do you have access to the following technologies for use as a peer support specialist? Check all that apply.

- Smartphones
- Tablets
- Desktop computers
- Laptop computers
- Routers and modems
- High speed internet
- Printers
- Windows Office 365 (e.g., Microsoft Word, Microsoft Excel)
- None

1. *[If respondent indicated that they are not a peer support specialist]* Do the peer support specialists that work in your organization have access to the following technologies for use as peer support specialists? Check all that apply.

- Smartphones
- Tablets
- Desktop computers
- Laptop computers
- Routers and modems
- High speed internet
- Printers
- Windows Office 365 (e.g., Microsoft Word, Microsoft Excel)
- None

*[For the following questions: questions were only given to respondents if they indicated the peer support specialists had access to the specific technology]*

1. Do the smartphones need to be updated to function appropriately?

- Yes
- No

1. Do the tablets need to be updated to function appropriately?

- Yes
- No

1. Do the desktop computers need to be updated to function appropriately?

- Yes
- No

1. Do the laptop computers need to be updated to function appropriately?

- Yes
- No

1. Do the routers and modems need to be updated to function appropriately?

- Yes
- No

1. Does the high-speed internet need to be updated to function appropriately?

- Yes
- No

1. Does Windows Office 365 (e.g., Microsoft Word, Microsoft Excel) need to be updated to function appropriately?
   - Yes
   - No

*Virtual recovery support services*

*[The following questions were given to the respondents if they indicated they are a peer support specialist.]*

1. Are you using any of these technologies to deliver virtual (i.e., through video, chat, discussion boards, etc.) recovery support services?
   - Yes
   - No
2. From the technologies you have access to from above, which ones are you using to deliver virtual recovery support services?

- Smartphones
- Tablets
- Desktop computers
- Laptop computers
- Routers and modems
- High speed internet
- Printers
- Windows Office 365 (e.g., Microsoft Word, Microsoft Excel)
- None

1. What virtual recovery support services platform are you using (e.g., RecoveryLink, Recovery Data Platform [RDP], Zoom, etc.)?

___________________________________

1. What do you like about this virtual recovery support service (e.g., frequency of meeting with participants, data collection on participant [i.e., How many times the participant accessed the service?], ease of connecting with participants, etc.)?

___________________________________

1. What do you not like about this virtual recovery support service (e.g., system crashes frequently while using it, current technology does not allow for easy use, the service does not collect enough information about the participant, etc.)?

___________________________________

1. Approximately, what proportion of your participants do you think are currently engaging in virtual recovery support services?

- 0-20%
- 21-40%
- 41-60%
- 61-80%
- 81-100%

*[The following questions were given to the respondents if they indicated they are not a peer support specialist.]*

1. Are the peer support specialists using any of these technologies to deliver virtual (i.e., through video chat, discussion boards, etc.) recovery support services?
   - Yes
   - No
2. From the technologies they have access to from above, which ones are the peers using to deliver virtual recovery support services?
   - Smartphones

- Tablets
- Desktop computers
- Laptop computers
- Routers and modems
- High speed internet
- Printers
- Windows Office 365 (e.g., Microsoft Word, Microsoft Excel)
- None

1. What virtual recovery support services are the peers using? (e.g., RecoveryLink, Recovery Data Platform [RDP], Zoom, etc.)

___________________________________

1. What do the peers like about this virtual recovery support service (e.g., frequency of meeting with participants, data collection on participant [i.e., How many times the participant accessed the service?], ease of connecting with participants, etc.)?

___________________________________

1. What do the peers not like about this virtual recovery support service (e.g., system crashes frequently while using it, current technology does not allow for easy use, the service does not collect enough information about the participant, etc.)?

___________________________________

1. Approximately, what proportion of your participants do you think are currently engaging in virtual recovery support services?

- 0-20%
- 21-40%
- 41-60%
- 61-80%
- 81-100%

*[All survey respondents if they answer ‘No’ to Question 10 (i.e., Are you using any of these technologies to conduct digital recovery support services?).]*

1. Approximately what proportion of your participants do you thinking would/could engage in virtual recovery support services?

- 0-20%
- 21-40%
- 41-60%
- 61-80%
- 81-100%

**S2. Semi-Structured Interview Guides**

**VERSION 1: Respondents/Organizations Reporting Using a D-RSS**

**Introduction**

Now for recording purposes, I want us all to introduce ourselves again. My name is [name], the time is [time], today is [date].

**Q.** Could you share your name, and the name of your organization again?

**Q.** To get started, could you share a bit more about the region and population you serve?

- Probe: What (geographical) area does your organization primarily cover?
- Probe: How wide is your coverage across surrounding communities?

**Section 1**

In this first part of the interview, we are hoping to learn more about your current “digital peer recovery support system” or D-RSS and how it functions for your organization.

I understand from your survey that your organization is currently using a D-RSS and that you are using the XYZ system. Is that correct? (If not, ask and update with correct D-RSS)

We’d like to know more about what you like/find useful in this system and what you don’t like/find less useful.

**Q.** Would you take a moment to talk with us about the features/capabilities of this system that you find most useful?

- Probe: What features do you find the most useful and why?
- Probe: What features do you find most helpful in your work?

**Q.** Would you now take a moment to talk with us about the features/capabilities of this system that you find less useful?

- Probe: What aspects of this system to you not like/find less useful/don’t use and why?
- Probe: What aspects of this system block your ability to do your job? Please describe.

We’re also interested in learning more about what barriers you faced in adopting your D-RSS, and about what helped you reduce the impact of those barriers.

**Q.** Would you take a moment to talk with us about/describe the barriers your organization faced in adopting your D-RSS?

**Q.** Would you also take a moment to describe what it took to remove or manage those barriers and enable success in using the D-RSS?

We’d like to learn more about what features you need but don’t have in your current D-RSS.

**Q.** Are there any features that your current D-RSS does not include that you wish it did include?

(Another way to ask this question) Have you used any other software in the past or know of another system that you believe is better suited to your needs?

- Probe: this could be anything regarding delivery or documentation of PRSS.  If yes, could you give us an example?

**Q.** If a D-RSS was available that included that/those wish list features/capabilities, how likely is it that you would switch to the new system?

- Probes: How difficult would it be for your organization to switch? What are the things that would make this transition difficult?

**Section 2**

In this next section, we have some questions regarding participant literacy (general and technological), access, and comfort with different modes of technology that we’d like to ask.

**Q.** Approximately, what proportion of your participants are fluent in English (reading and writing)?

- Probe: If not English, what other primary languages do your participants speak?

**Q.** Approximately, what proportion of your participants are experiencing homelessness?

**Q.** Approximately, what proportion of your participants cannot read/have significant difficulty reading?

We would also like to know more about the access that your participants have to technological tools or equipment.

**Q.** What technological tools or equipment do your participants have access to? (If only a few mentioned, could probe with the rest of the tools listed above: computers, smartphones, tablets, internet, reliable cell phone coverage)

- Probe: What technological tools or equipment do your participants not have access to?

**Q.** What proportion of your participants have access to these tools?

- Probe: What proportion of your participants don’t have access to these tools?

In the survey you responded to, you mentioned that [answer]% of your participants were enrolled in DRSS.

**Q.** How do these tools, that we talked about before, impact the participants’ access to DRSS? (If needed, an example could be bad cell phone coverage, and the inability to log into a DRSS platform)

- Probe: How does the engagement with DRSS look like for these participants?
- Probe: For the participants that are not enrolled in DRSS, what would facilitate access to DRSS?
- Probe: What kind of training do your participants get before accessing DRSS?

**Q.** I now would like to list again some technological actions or tools that people might do or use. In a scale of 1 to 10, with one being ‘not comfortable at all’ and 10 being ‘extremely comfortable, I’d like to know how comfortable **your** **participants** are with these actions or tools (list each bullet point):

[NOTE: the choices below are prompts for the interviewers, could be more or less, or different, depending on conversation and other responses]:

- Navigating to websites on computers
- Navigating to websites on tablets or smartphones
- Installing an application onto a smartphone
- Apple iPhones
- Android smartphones
- Video conferencing (e.g., Zoom, Skype, Microsoft Teams)
- Text messaging
- Phone calls

**Q.** Now, using the same scale, with one being ‘not comfortable at all’ and 10 being ‘extremely comfortable, I’d like to know how comfortable you/your **peer recovery support specialists** are with these actions or tools (list each bullet point):

[NOTE: the choices below are prompts for the interviewers, could be more or less, or different depending on conversation and other responses]:

- Navigating to websites on computers
- Navigating to websites on tablets or smartphones
- Installing an application onto a smartphone
- Apple iPhones
- Android smartphones
- Video conferencing (e.g., Zoom, Skype, Microsoft Teams)
- Text messaging
- Phone calls

**Q.** What kind of training do your peer recovery support specialists get before providing DRSS?

**Q.** In your opinion, what is the biggest barrier to using a digital peer recovery support service?

**Q.** What might be helpful in overcoming this barrier?

**Q.** Before we finish this interview, is there anything that I haven’t asked that you think is important for me to know?

**VERSION 2: Respondents/Organizations Reporting Not Using a D-RSS**

**Introduction**

Now for recording purposes, I want us all to introduce ourselves again. My name is [name], the time is [time], today is [date].

**Q.** Could you share your name, and the name of your organization again?

**Q.** To get started, could you share a bit more about the region and population you serve?

- Probe: What (geographical) area does your organization primarily cover?
- Probe: How far out do you reach to other surrounding communities?

**Section 1**

In this first part of the interview, we are hoping to learn more about your needs if you were to adopt a D-RSS in your organization.

I understand from your survey that your organization is currently not using a D-RSS. We’d like to know more about your thoughts on a D-RSS and what you would like/might find useful in a D-RSS.

**Q.** What is your current understanding of what a D-RSS is and what it offers?

**Q.** Is this something that would be useful in your organization? Why? /Why not?

We’re also interested in learning about what barriers you might face in adopting a D-RSS.

**Q.** Based on your knowledge, what do you think might be the biggest barriers your organization would face in adopting a D-RSS?

**Q.** As you think about these barriers, what might help remove or reduce the impact of these barriers and enable success in using a D-RSS?

We’d like to learn more about what features you would really need/want in a D-RSS.

**Q.** Are there any features that your organization would need or really want in a D-RSS?

- Probe: this could be anything regarding delivery or documentation of RSS.

**Q.** If a D-RSS was available that included that/those wish list features/capabilities, how likely is it that you would adopt this system?

- Probes: How difficult would it be for your organization to adopt a D-RSS? What are the things that would make this transition difficult?

**Section 2**

In this next section, we have some questions regarding participant literacy (general and technological) and comfort with different modes of technology that we’d like to ask.

**Q.** Approximately, what proportion of your participants are fluent in English (reading and writing)?

- Probe: If not English, what other primary languages do your participants speak?

**Q.** Approximately, what proportion of your participants are experiencing homelessness?

**Q.** Approximately, what proportion of your participants cannot read/have significant difficulty reading?

We would also like to know more about the access that your participants have to technological tools or equipment.

**Q.** What technological tools or equipment do your participants have access to? (If only a few mentioned, could probe with the rest of the tools listed above: computers, smartphones, tablets, internet, reliable cell phone coverage)

- Probe: What technological tools or equipment do your participants not have access to?

**Q.** What proportion of your participants have access to these tools?

- Probe: What proportion of your participants don’t have access to these tools?

In the survey you responded to, you mentioned that [answer]% of your participants could be interested in enrolling in DRSS.

**Q.** How would these tools, that we talked about before, impact the participants’ access to DRSS? (If needed, an example could be bad cell phone coverage, and the inability to log into a DRSS platform)

- Probe: How would the engagement with DRSS look like for these participants?
- Probe: What would facilitate access to DRSS?
- Probe: What kind of training would your participants need before accessing DRSS?

**Q.** I now would like to list again some technological actions or tools that people might do or use. In a scale of 1 to 10, with one being ‘not comfortable at all’ and 10 being ‘extremely comfortable, I’d like to know how comfortable **your** **participants** are with these actions or tools (list each bullet point):

[NOTE: the choices below are prompts for the interviewers, could be more or less, or different depending on conversation and other responses]:

- Navigating to websites on computers
- Navigating to websites on tablets or smartphones
- Installing an application onto a smartphone
- Apple iPhones
- Android smartphones
- Video conferencing (e.g., Zoom, Skype, Microsoft Teams)
- Text messaging
- Phone calls

**Q.** Now, using the same scale, with one being ‘not comfortable at all’ and 10 being ‘extremely comfortable, I’d like to know how comfortable you/your **peer recovery support specialists** are with these actions or tools (list each bullet point):

[NOTE: the choices below are prompts for the interviewers, could be more or less, or different depending on conversation and other responses]

- Navigating to websites on computers
- Navigating to websites on tablets or smartphones
- Installing an application onto a smartphone
- Apple iPhones
- Android smartphones
- Video conferencing (e.g., Zoom, Skype, Microsoft Teams)
- Text messaging
- Phone calls

**Q.** What kind of training do your peer recovery support specialists get before providing DRSS?

**Q.** In your opinion, what would be the biggest barrier to using a digital peer recovery support service?

- Probe: What would be the biggest barrier for your participants?
- Probe: What would be the biggest barrier for you/your organization?

**Q.** What might be helpful in overcoming this barrier?

**Q.** Before we finish this interview, is there anything that I haven’t asked that you think is important for me to know?
